# Narcolepsy Type 1 Is Associated with a Systemic Increase and Activation of Regulatory T Cells and with a Systemic Activation of Global T Cells

**DOI:** 10.1371/journal.pone.0169836

**Published:** 2017-01-20

**Authors:** Michel Lecendreux, Guillaume Churlaud, Fabien Pitoiset, Armelle Regnault, Tu Anh Tran, Roland Liblau, David Klatzmann, Michelle Rosenzwajg

**Affiliations:** 1 AP-HP, Pediatric Sleep Center and National Reference Centre for Orphan Diseases, Narcolepsy, Idiopathic Hypersomnia and Kleine-Levin Syndrome (CNR narcolepsie-hypersomnie), CHU Robert-Debré, Paris, France; 2 Pediatric Sleep Disorders Center, Robert Debré Hospital, Paris, France; 3 AP-HP, Hôpital Pitié-Salpêtrière, Biotherapy (CIC-BTi) and Inflammation-Immunopathology-Biotherapy Department (I2B), Paris, France; 4 Sorbonne Université, UPMC Univ Paris 06, UMRS 959, Immunology-Immunopathology- Immunotherapy (I3), Paris, France; 5 INSERM, UMR_S 959, Immunology-Immunopathology-Immunotherapy (I3), Paris, France; 6 Aviesan/Institut Multi-Organismes Immunologie, Hématologie et Pneumologie (ITMO IHP), Paris, France; 7 Pediatrics department, Centre hospitalo-universitaire de Nîmes, 30029 Nîmes Cedex 9, France. INSERM U1012, Le Kremlin Bicêtre, France; 8 INSERM UMR1043—CNRS UMR5282—Université Toulouse III, Toulouse, France; IMAGINE, FRANCE

## Abstract

Narcolepsy is a rare neurologic disorder characterized by excessive daytime sleepiness, cataplexy and disturbed nocturnal sleep patterns. Narcolepsy type 1 (NT1) has been shown to result from a selective loss of hypothalamic hypocretin-secreting neurons with patients typically showing low CSF-hypocretin levels (<110 pg/ml). This specific loss of hypocretin and the strong association with the HLA-DQB1*06:02 allele led to the hypothesis that NT1 could be an immune-mediated pathology. Moreover, susceptibility to NT1 has recently been associated with several pathogens, particularly with influenza A H1N1 virus either through infection or vaccination. The goal of this study was to compare peripheral blood immune cell populations in recent onset pediatric NT1 subjects (post or non-post 2009-*influenza* A H1N1 vaccination) to healthy donors. We demonstrated an increased number of central memory CD4^+^ T cells (CD62L^+^ CD45RA^-^) associated to an activated phenotype (increase in CD69 and CD25 expression) in NT1 patients. Percentage and absolute count of regulatory T cells (Tregs) in NT1 patients were increased associated with an activated phenotype (increase in GITR and LAP expression), and of activated memory phenotype. Cytokine production by CD4^+^ and CD8^+^ T cells after activation was not modified in NT1 patients. In H1N1 vaccinated NT1 patients, absolute counts of CD3^+^, CD8^+^ T cells, and B cells were increased compared to non-vaccinated NT1 patients. These results support a global T cell activation in NT1 patients and thus support a T cell-mediated autoimmune origin of NT1, but do not demonstrate the pathological role of H1N1 prophylactic vaccination. They should prompt further studies of T cells, particularly of Tregs (such as suppression and proliferation antigen specific assays, and also T-cell receptor sequencing), in NT1.

## Introduction

Narcolepsy type 1 (NT1) is a rare neurological disease that affects 1 per 2000 individuals. It is a disabling chronic sleep disorder that disturbs quality of life. NT1 is characterized by excessive daytime sleepiness, sleep paralysis, hypnagogic hallucinations and cataplexy, which are sudden episodes of muscle weakness triggered by emotional factors. Although not always present, cataplexy is highly specific to NT1 and represents an important clinical marker of this condition. NT1 is caused by the loss of hypothalamic hypocretin/orexin-producing neurons [1] with a decreased concentration of hypocretin in cerebrospinal fluid [2]. These neurons are involved in the regulation of sleep-wakefulness [3,4]. To date, the cause of this neuronal loss remains unknown, but several assumptions are made, particularly in relation with its immune origin.

NT1 is strongly associated with specific human leukocyte antigen alleles (HLA) since 95% of NT1 patients with cataplexy carry the HLA-DRB1*15:01/DQB1*06:02 haplotype [5] compared with 25% of the general population [6]. HLA-DPB1*05:01 also confers a risk of NT1 whereas the HLA-DPA1*01:03 and DPB1*04:02 alleles seem to be protective [7]. HLA class II alleles are thus strongly associated with susceptibility to NT1. Furthermore, some polymorphisms at the T cell receptor (TCR) alpha locus are now considered as NT1 susceptibility genes [8,9]. Molecules modulating directly T cell functions as OX40L [10] or survival such as P2RY11 [11] are additional parameters showing the involvement of the immune system in triggering NT1 [12].

Some possible immunological triggers like the pandemic 2009 influenza H1N1 virus [13], whether after vaccination [14,15] or direct H1N1 seasonal infection [16], or *Streptococcus pyogenes* infections [17,18] were reported to be associated with NT1 occurrence. Indeed, in China, the onset of NT1 in children follows seasonal peaks, with increases after winter-related infections [16]. Epidemiologic investigations in China and in several European countries have revealed an association between NT1 and anti-*influenza* A immune response in relation with influenza infection or vaccination [15,16]. Furthermore, a recent study associated HLA-DQ variants with age of onset NT1 following the 2009 H1N1 *influenza* pandemic in China [19]. The results of these studies are in favor of an autoimmune origin of the disease: exposure to specific pathogens or antigens could generate and select hypocretin-specific immune cells [20,21], triggering NT1. The possible role of the different adjuvants contained in A H1N1 pandemic vaccine in triggering NT1 has been proposed [22] but is still debated since an increase of narcolepsy cases has been observed in country were adjuvants were not used [22]. For now, there is little evidence showing an autoimmune origin of NT1 but Ahmed *et al* have recently shown that the Pandemrix flu vaccine triggers antibodies that can bind to the hypocretin receptor 2 in brain cells that help regulate sleepiness [23,24].

In most autoimmune diseases (AIDs), there is an imbalance between harmful self-specific effector T cells (Teffs) that attack normal tissues and regulatory T cells (Tregs) that normally control them. Tregs are essential players in the control of all immune responses, including responses to self, tumors, and infectious agents [25], and in the control of autoimmune and inflammatory disorders [26,27]. Treg population has been shown to play an important role in the maintenance of peripheral tolerance [28].

In this study, we investigated peripheral blood immune cell populations in recent onset pediatric NT1 subjects (post or non-post 2009-*influenza* A H1N1 vaccination) and whether Tregs could play a role in NT1 occurrence. Blood lymphocytes subsets were phenotyped in depth by flow cytometry to determine whether NT1 could be associated with quantitative or qualitative abnormalities of Treg cells or of other lymphocytes subsets as detected in other AIDs [29,30].

## Materials and Methods

### Patients characteristics

Patients were recruited from the reference center for pediatric narcolepsy at Robert-Debré Hospital (Paris, France). The study population consisted of pediatric and young adults patients under the age of 21 years old fulfilling the international criteria for NT1 (as defined in the International Classification of Sleep Disorders, ICSD-3). Patients were seen close to onset of their first symptoms. NT1 patients were divided in two groups: post H1N1 vaccination NT1 patients and NT1 patients unrelated to H1N1 vaccination. Post-H1N1 NT1 patients have been defined as NT1 patients vaccinated with Pandemrix vaccine before disease onset. Both NT1 groups were compared to healthy donors (HD). Patients and HD were enrolled in the Narcobank study. We obtained written informed consent from the next-of-kin, caretakers, or guardians on behalf of the minors/children enrolled in the Narcobank study. An information note for a minor patient was explained and given to the minors / children enrolled in our study. Narcobank aims to study biomarkers and genetic risk factors of narcolepsy and other rare central hypersomnias [31]. The protocol of Narcobank was approved by the research scientific committee of the ANSM [31], and our specific study (immunomonitoring of NT1 patients) has been approved by our Local Ethics Committee (Committee for protection of people CPP of Ile-de-France VI). According to French law, this biological collection was registered to the Ministry of higher education and research (Number DC- 2008–417).

### Immunomonitoring

Peripheral blood samples were collected by venipuncture in heparinized tubes. Because of the limited amount of blood collected, all phenotypic markers and cytokine assays were only investigated for some patients. Peripheral blood mononuclear cells (PBMCs) were isolated from blood samples using Ficoll-Hypaque density gradient centrifugation. PBMCs were washed twice in phosphate buffered saline (PBS) containing 2% foetal calf serum and numerated. Whenever possible, cells were frozen for further investigations.

#### Flow cytometry

Flow cytometry was performed according to previously published routine methods used at the Pitié-Salpêtrière Biotherapy Department [32]. The absolute counts of lymphocytes subsets (CD3^+^, CD4^+^, CD8^+^ T lymphocytes, Tregs, CD19^+^ B lymphocytes and CD3^-^CD56^+^ NK cells) were established from fresh blood samples using CYTO-STAT tetraCHROME kits with Flowcount fluorescent beads as internal standard and tetra CXP software with a FC500 cytometer according to manufacturer’s instructions.

PBMCs were labeled with mAbs directly conjugated either to Fluorescein isothiocyanate (FITC), Phycoerythrin (PE), Phycoerythrin-Texas Red (ECD), Allophycocyanin (APC), phycoerythrin-Cyanyn 7 (PE-Cya7), APC–Alexafluor 700 (APCa700) or APC–Alexafluor 750 (APC-a750) were used for: CD3-APCa750, CD4-ECD, CD8-APCa700, CD19-ECD, CD45RO-FITC, CD56-PCy7 and CD152-PE all from Beckman Coulter (Villepinte, France). CD25-PE, CD45RA-APC and CD45RA-PCy7 were from BD Biosciences. CD127-FITC was from eBioscience. CD25-APC and LAP-PE were from R&D Systems and GITR-PE from Miltenyi. Matched mouse isotype control antibodies were used. The gating strategies are presented in S1 and S2 Figs.

In our study, we decided not to use FoxP3 staining to define regulatory T cells (Tregs) as in our clinical laboratory use we have a perfect correlation between Tregs characterized as CD4^+^CD25^high^ FoxP3^+^ T cells and Tregs characterized as CD4^+^CD25^high^ CD127^-/lo^ T cells (S3 Fig).

Cells acquisition and analysis by flow cytometry were performed using a Navios Cytometer (Beckman Coulter). Instrument setting parameters (gains, compensations, and threshold) were set with machine software (Navios Software; Beckman Coulter) in conjunction with calibration beads (Flow-set beads, Cytocomp kit, and CYTO-TROL Control Cells; Beckman Coulter). Machine reproducibility was verified with standardized beads (Flow-check; Beckman Coulter). Data were analyzed with Kaluza software (Beckman Coulter).

#### Intracellular cytokines

Total PBMCs were stimulated with 1 μg/mL phorbol 12-myristate 13- acetate (PMA) (Sigma-Aldrich) and 0.5 μg/mL ionomycin (Sigma-Aldrich) for 4 hours in the presence of 1 μl/mL GolgiPlug (BD) in RPMI 1640 medium supplemented with 5% of serum blood type AB, 2 mmol/L L-glutamine, 100 U/mg/mL penicillin/streptomycin, and at 5x10^4^ cells/well. After cell surface staining, intracellular staining with IFN-gamma, TNF-alpha, IL-10 and IL-4 was performed using the Cytofix/Cytoperm kit (BD). Cells were acquired on Navios Cytometer (Beckman Coulter) and analyzed with Kaluza software (Beckman Coulter).

#### Plasma cytokine measurement

Plasma samples were collected, aliquoted and stored at -80°C until analyzed. Quantitative determination of 6 cytokines (IFN-gamma, IL-1RA, IL-6, IL-8, IL-10 and TNF-alpha) was performed using Human Milliplex kits (Millipore) in accordance with the manufacturer protocols.

### Statistical analysis

Statistical significances between patients groups were evaluated using GraphPad Prism version 5.00 for Windows (GraphPad Software, San Diego, CA, http://www.graphpad.com) and calculated using either unpaired t test (if normal distribution using D’Agostino and Pearson omnibus normality test, alpha = 0.05) or Mann-Whitney test (comparison of means, non-parametric unpaired test, two-tail *P* value), with *P* < 0.05 (*) taken as statistical significance (** *P* < 0.01, *** *P* < 0.001, NS = non-significant).

## Results

### Patients’ characteristics

The study population consisted of 31 NT1 patients: 17 non H1N1 and 14 H1N1 (Table 1). Both groups were compared to 32 HD with similar age and gender characteristics. All 31 NT1 patients were HLA-DQB1*06:02 positive and exhibited cataplexy. Of the 21 patients tested for hypocretin levels, hypocretin deficiency was found in 20. Regarding the NT1 disease duration, median duration was 591 days. Shorter disease duration was observed in H1N1 (487 days) NT1 patients as compared to non H1N1 (1295 days) patients (p = 0.002).

**Table 1:** Demographic and clinical parameters of healthy donors (HD) and H1N1 or non H1N1 NT1 patients.

Demographics	HD (n = 32)	NT1 (n = 31)	Non H1N1 NT1 (n = 17)	H1N1 NT1 (n = 14)
**Age (years)** ^**a**^	13 (4–17)	13 (5–21)	13 (6–21)	13 (5–16)
**Sex ratio M/F**	17/15	18/13	12/5	7/7
**Disease duration (days)** ^**a**^	-	591 (169–3376)	1295 (169–3376)	487 (199–622)
HLA-DQB1*06:02 + ^**b**^	-	31 / 31 (100%)	17 / 17 (100%)	14 / 14 (100%)
**Hypocretin levels < 110 pg/mL** ^**b**^	-	20 / 21 (95%)	7 / 7 (100%)	13 / 14 (93%)
**Cataplexy +** ^**b**^	-	31 / 31 (100%)	17 / 17 (100%)	14 / 14 (100%)

^a^Continuous variables were expressed by n; median and range (min-max).

^b^Variables were expressed by number of positive out of number of tested and percentage (%).

### Absolute counts of the main lymphocyte subsets

There were no significant changes in CD3^+^, CD4^+^, CD8^+^ T cell, B cell and NK cell numbers in peripheral blood between HD and NT1 patients (Table 2). Tregs absolute count in blood was significantly increased in NT1 patients (38 ± 4 cells / mm^3^ in HD vs 51 ± 4 cells / mm^3^ in NT1, p = 0.003). Regarding the differences in lymphocyte subsets absolute counts between H1N1 and non H1N1 NT1 patients, CD3^+^ and CD8^+^ T cells were found significantly increased in H1N1 group compared to non H1N1 (Table 2).

**Table 2:** Absolute lymphocyte counts of HD and NT1 patients.

Cell type	HD (n = 32)	NT1 (n = 28)	p^a^	Non H1N1 NT1 (n = 15)	H1N1 NT1 (n = 13)	p^b^
**CD3**^**+**^ **T cells**	1428 ± 75	1503 ± 110	NS	1267 ± 62	1774 ± 204	* 0.018
**CD4**^**+**^ **T cells**	823 ± 43	875 ± 62	NS	778 ± 44	987 ± 119	NS
**CD8**^**+**^ **T cells**	486 ± 35	508 ± 60	NS	399 ± 34	633 ± 115	* 0.048
**Tregs**	38 ± 4	51 ± 4	** 0.003	50 ± 5	52 ± 7	NS
**CD19**^**+**^ **B cells**	370 ± 34	363 ± 26	NS	332 ± 29	397 ± 43	NS
**CD3**^**-**^ **CD56**^**+**^ **NK cells**	154 ± 12	173 ± 21	NS	170 ± 23	176 ± 37	NS

Results are expressed as mean ± SEM (cells / mm^3^). p^a^ represents the statistical differences between HD and NT1, and p^b^ between H1N1 and non H1N1 NT1 patients; statistical differences are assessed by Unpaired t test (when normality test passed using D’Agostino and Pearson omnibus normality test, alpha = 0.05) or Mann-Whitney test.

### Global T cell activation in narcolepsy

**Fig 1:**
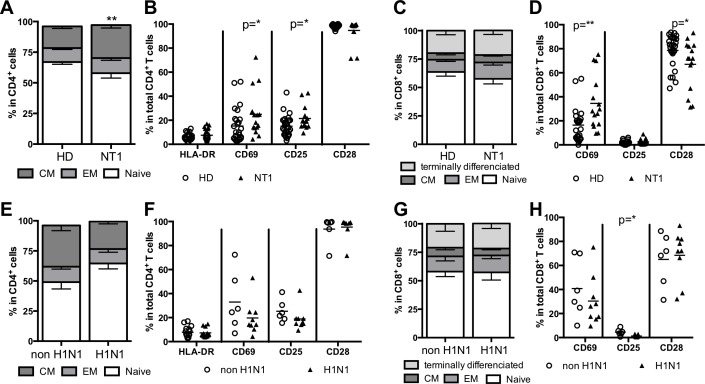
CD4^+^ and CD8^+^ T cell differentiation and activation phenotypes according to NT1 and H1N1 status. Central memory (CM), effector memory (EM) and naïve CD4^+^ and CD8^+^ T cells in peripheral blood were characterized for HD and NT1 patients (A and C) and for H1N1 or non H1N1 NT1 patients (E and G). Activation markers for CD4^+^ and CD8^+^ T cells in peripheral blood were characterized for HD and NT1 patients (B and D) and for H1N1 or non H1N1 NT1 patients (F and H).

Regarding CD4^+^ T cell differentiation, we observed an increased frequency of central memory CD45RA^-^ CD62L^+^ CD4^+^ T cells in NT1 patients compared to HD (17.7 ± 1.7% in HD vs 26.9 ± 2.4% in NT1, p = 0.001) whereas naïve CD45RA^+^ CD62L^+^ CD4^+^ T cells and CD45RA^-^ CD62L^-^ CD4^+^ effector memory frequencies were not significantly different (Fig 1A). CD4^+^ T cells were then examined for expression of activation markers i.e., CD25, CD69, and HLA-DR, and for expression of CD28 (functionality marker) (Fig 1B). In NT1 patients, there was increased expression of CD25 (15.0 ± 1.5% in HD vs 21.5 ± 2.5% in NT1, p = 0.038) and CD69 (15.2 ± 2.7% in HD vs 25.0 ± 5.0% in NT1, p = 0.045) whereas HLA-DR and CD28 expression was comparable to HD (Fig 1B).

Regarding CD8^+^ T cell differentiation, naïve and memory frequencies were not modified in NT1 compared to HD (Fig 1C). In CD8^+^ T cells of NT1 patients, CD69 expression was increased (16.5 ± 3.0% in HD vs 34.5 ± 5.7% in NT1, p = 0.003) whereas CD28 expression was decreased compared to CD8^+^ T cells of HD (78.7 ± 2.6% in HD vs 67.1 ± 5.3% in NT1, p = 0.032) (Fig 1D).

No influence of the H1N1 status on CD4^+^ and CD8^+^ T cell differentiation and activation phenotypes of NT1 patients was found here, except for a decreased expression of CD25 in CD8^+^ T cells in H1N1 patients (4.5 ± 1.1% in non H1N1 vs 1.3 ± 0.3% in H1N1, p = 0.012) (Fig 1E–1H).

Frequencies of naïve (IgD^+^CD27^-^), memory (IgD^-^CD27^+^) and marginal zone (IgD^+^CD27^+^) CD19^+^ B cells were also studied (S4A and S4D Fig). No differences were detected between HD and NT1 patients (S4A Fig), or between H1N1 and non H1N1 NT1 patients (S4D Fig). No differences were found in the frequency of NK cells (S4B and S4E Fig) and TCR γδ T cells (S4C and S4F Fig) between HD and NT1 patients, or between H1N1 and non H1N1 NT1 patients.

### Modulation of plasma cytokine profiles in NT1

**Fig 2:**
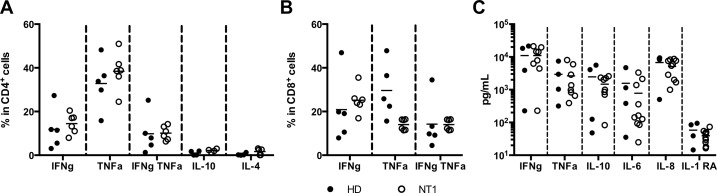
**Cytokines profiles after PMA/ionomycine activation** in CD4^+^ (A) and CD8^+^ (B) T cells or directly in plasma (C) of HD (n = 4–5) and NT1 patients (n = 6–8).

A panel of 6 cytokines was measured to investigate whether NT1, associated or not to H1N1, could be associated to a specific Th1/Th2 cytokines profile. Cytokines were first assayed by intracellular staining on total PBMCs after 4 hours of PMA-ionomycin activation. After activation, a fair proportion of CD4^+^ and CD8^+^ T cells produced IFN-gamma and TNF-alpha, whereas nearly no IL-10 and IL-4 were detected in CD4^+^ T cells (Fig 2A and 2B). About 10–15% of CD4^+^ and CD8^+^ T cells secreted both IFN-gamma and TNF-alpha cytokines. No statistical differences regarding the frequency of cytokine-producing CD4^+^ (Fig 2A) and CD8^+^ (Fig 2B) T cells between HD and NT1 patients were detected, although the small sample size limits sensitivity.

Cytokine levels were also assessed in plasma of HD and NT1 patients (Fig 2C). IFN-gamma, TNF-alpha, IL-10, IL-6 and IL-8 were detected in both groups, whereas IL-1 RA was nearly undetectable. No statistical differences were shown for all cytokine levels between HD and NT1 patients.

### Increased frequency of Treg in NT1, associated with an activated phenotype

**Fig 3:**
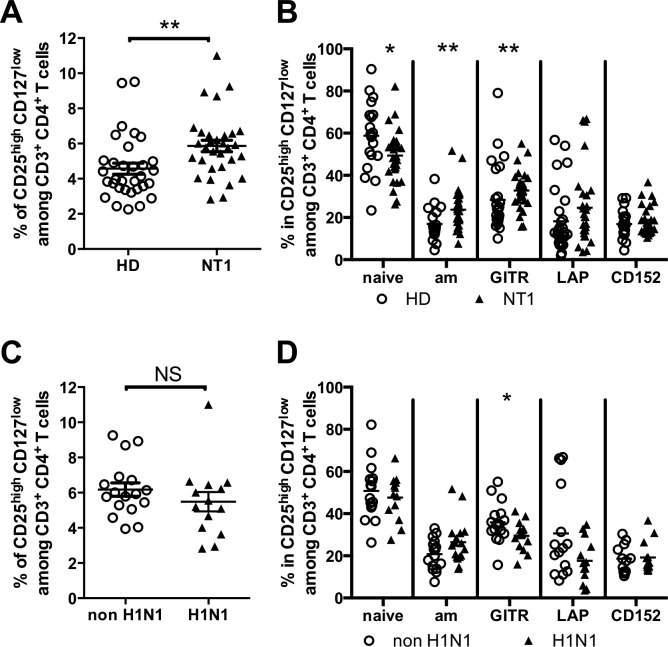
**Tregs percentages and phenotype according to NT1 (A-B) and to H1N1 (C-D) status.** 32 HD, 31 NT1 patients (including 14 H1N1 and 17 non H1N1 NT1 patients), were assayed for Tregs phenotypes.

Because Treg population was shown to play an important role in the maintenance of peripheral tolerance, we measured the frequency of Tregs (characterized here as CD4^+^CD25^high^ CD127^-/lo^ T cells) (S3 Fig) in the blood of NT1 patients and HD. The frequency of Tregs in HD was 4.6 ± 0.3%. Interestingly, NT1 patients showed a significant increased frequency of Treg (5.9 ± 0.3%, p = 0.0014) compared to controls (Fig 3A). Expression of molecules associated with Treg function or differentiation was then investigated (Fig 3B). GITR (28.4 ± 3.1% in HD vs 32.8 ± 1.6% in NT1, p = 0.0097) expression was increased on Tregs whereas LAP (membrane TGF-beta) and CD152 (CTLA-4) expression was unchanged. Tregs from NT1 patients displayed an activated memory phenotype (defined as HLA-DR^+^ CD45RA^-^) (17.0 ± 1.9% in HD vs 23.7 ± 1.6% in NT1, p = 0.007), with a decreased percentage of HLA-DR^-^ CD45RA^+^ naïve Tregs (58.8 ± 3.6% in HD vs 49.3 ± 2.3% in NT1, p = 0.024) compared to HD. In NT1 patients, the increased frequency of Tregs and their activated memory phenotype were not correlated to NT1 duration or to age (S5 Fig).

Treg percentages (5.5 ± 0.5% vs 6.2 ± 0.4%) and activation phenotypes were not statistically different between H1N1 and non H1N1 NT1 patients respectively, whereas a decreased expression of GITR was observed in H1N1 patients (35.6 ± 2.4% in non H1N1 vs 29.4 ± 1.7% in H1N1, p = 0.041) (Fig 3C and 3D).

## Discussion

The specific loss of hypocretinergic neurons in NT1 is thought to result from an autoimmune attack [12,24]. This hypothesis is supported by evidence of both environmental and genetic factors pointing toward an involvement of the immune system. Indeed, recent advances in the identification of susceptibility genes (such as HLA locus) and environmental exposures (pandemic *influenza* 2009 vaccination) [22] provide strong support that NT1 is an immune-mediated disease. Although the exact mechanism of hypocretin deficiency is still missing, evidence strongly favors an immune-mediated or autoimmune etiology. Liblau et al [12] and Degn M et al [33] proposed a T cell-mediated pathogenesis of NT1 targeting specifically hypocretin neurons in genetically predisposed individuals. For now there is no proof for a T cell-mediated autoreactivity to hypocretin or another antigen presents in brain that could be presented by HLA molecules associated with NT1 (such as DQB1*06:02) and that could explain disease pathogenesis. Nevertheless, in addition to the association with specific HLA molecules, genetic association with the T-cell receptor α [8] and β [19] loci to narcolepsy predisposition has been demonstrated, emphasizing the supposed role of T cells in the pathophysiology of NT1. These specific HLA-TCR interactions found in NT1 support the hypothesis that a T-cell mediated autoimmune attack causes the specific degeneration of hypocretin neurons [34]. Supporting the hypothesis of an autoimmune origin of NT1, autoantibodies to Tribbles homolog 2, which is expressed by hypocretin neurons but also largely in the brain, have been detected in some NT1 patients [35], and passive transfer of these autoantibodies could mimic NT1 in mice [36]. Furthermore Ahmed et al. found, in Pandemrix vaccinated patients, antibodies to *influenza* nucleoprotein cross-reacting with human hypocretin receptor 2, which could explain the narcolepsy associated with Pandemrix vaccination [24]. Finally, in the same direction, Saariaho et al. described autoantibodies against ganglioside GM3 associated to narcolepsy after Pandemrix vaccination [37].

The aim of our study was not to evaluate an antigen-specific immune response, but rather to evaluate the overall immune system activation. Patients with NT1 displayed blood phenotypic changes characterized by increased frequency and absolute count of CD4^+^ Tregs that were more activated, associated with increased levels of activated and memory effector CD4^+^ T cells. This activated phenotype and this change in Treg frequency have not been reported before and could be induced in reaction to an autoimmune process in NT1. Fontana et al. [38] have suggested that the immune-mediated destruction of hypocretin-producing neurons may be mediated by microglia/macrophages that become activated either by autoantigen specific CD4^+^ T cells or superantigen stimulated CD8^+^ T cells. Moreover, Kornum et al. [11] have shown the importance of T cells in NT1 pathology: they described polymorphisms of P2RY11 associated to NT1 having an effect on NK, CD8^+^ and CD4^+^ T cell viability. P2RY11 acts as an important regulator of immune-cell survival, and would have implications in NT1 by increasing T cell survival. These results are in line with our results showing a global activation of T cells. However, HDs were not HLA matched controls (they were not HLA typed), which is a limitation of our study.

Beside a global increase of CD8^+^ T cells that could be related to H1N1 vaccination, H1N1 impact on immune cell populations in NT1 patients is weak in our study. Pizza et al. showed no impact of H1N1 on the clinical picture of narcoleptic children, which was similar in post-vaccine and pre-H1N1 infection[39].

### Possible implication of tregs in triggering NT1

A key question raised by our study focuses on the role of Tregs in NT1. Here, Treg percentages and absolute counts are significantly increased in NT1 patients compared to HD (Table 2 and Fig 3). Treg frequencies found in HD and NT1 patients in our study were correlated with previous published results in children with other pathologies [40–43]. We suggest here that the global but weak inflammation in NT1 leads to an activation of all T cells, including Tregs, but a qualitative defect, either in these Tregs, or the in pathogenic immune cells, could prevent Tregs from maintaining peripheral tolerance. Indeed, a qualitative defect of Tregs has previously been described in human AIDs such as type 1 diabetes [29,44,45]. Long and Buckner [30] highlighted the need to better understand Treg plasticity and function in the context of autoimmunity. An increase in Tregs can be found in peripheral blood and in tissues affected by autoimmunity (rheumatoid arthritis and psoriasis for example) without full control of the autoimmune process [29,46–50]. Dalla Libera D. *et al*. also confirmed in acute multiple sclerosis (MS) an upregulation of the Treg compartment compared to stable MS [51]. According to the authors, Tregs’ increase is an attempt to dampen inflammation and to restore homeostasis. Impaired Treg-mediated suppression can occur in autoimmunity. First, defects in suppression may occur due to Treg-intrinsic defects. Secondly, effector T cells can become resistant to regulation, a phenomenon particularly true for Th17 effector cells. Finally, an altered microenvironment with, for example, altered antigen presenting cells functions or increase of pro-inflammatory cytokines in the environment can increase resistance to regulation. In our study we did not find any modifications in cytokines secretions between HD and NT1 patients. Nevertheless several studies showed disturbed productions of cytokines associated to NT1. Ambati et al. [17] showed that IFN-gamma production was significantly increased in blood from NT1 patients in response to streptococcus serotype M6 and streptodornase B protein antigenic stimulation. Chen et al. [52] showed increased TNF-alpha level in NT1 patients, and suggested that chronic inflammation has led to NT1. Furthermore, in a much larger study on cytokines/chemokines release, we described an increased stimulation of the immune system with high release of several pro- and anti- inflammatory serum cytokines and growth factors associated with NT1 [53]. Some of these increases were only significant when close to disease onset. In our study the average disease duration of NT1 is 562 days, which could explain the difference with our previous study where disease duration was 1 year. Probably due to the small number of patients tested, we did not find any increased production of pro-inflammatory cytokines (Fig 2), nevertheless a trend was observed with an increased production of IFN-gamma by CD4^+^ and CD8^+^ T cells after polyclonal activation in NT1 patients. Thus, as described in other studies [54,55], low-grade chronic inflammation is present in NT1 patients. Altered antigen presentation could also lead to Tregs defective functions. Polymorphisms in TCR alpha and beta loci have been associated to NT1 occurrence [8,19]. In NT1 patients, altered Treg TCR repertoire with lower polyclonality levels could be envisioned as a cause of tolerance loss. Using TCR deep sequencing, identification of dominant TCRs and antigen-specific Treg signatures, at steady state and during disease progression, will contribute to a better understanding of the pathophysiology of AIDs. Preliminary results in our laboratory suggested major differences in TCR repertoire composition and diversity between nTregs and amTregs in non-manipulated mice [56]. As narcolepsy is about to be classified as an immune-mediated disease [34] deep sequencing of NT1 T cell subsets should be considered.

In summary, low-grade inflammation is present in close to onset NT1 patients. This chronic inflammation is acting particularly on T cells, including Tregs. Functional assays should be performed in order to characterize a possible functional defect of Tregs (such as suppression and proliferation antigen specific assays, using H1N1 peptides, as molecular mimicry has been hypothesized between H1N1 virus and hypocretin-producing neurons [34] and such as TCR sequencing).

## Supporting Information

S1 Fig Representative flow cytometry analysis of CD4^+^ and CD8^+^ T cells and of CD4^+^ Tregs from human fresh heparinized peripheral blood. CD4^+^ and CD8^+^ T cells were gated from the lymphocytes gate. Naïve T cells were defined as CD45RA^+^ CD62L^+^ cells, central memory (CM) T cells as CD45RA^-^ CD62L^+^, effector memory (EM) T cells as CD45RA^-^ CD62L^-^, and terminally differentiated (TEMRA) T cells as CD45RA^+^ CD62L^-^. Activated memory Tregs (AM) were defined as CD45RA^-^ HLA-DR^+^.
(PDF)

S2 Fig Representative flow cytometry analysis of B lymphocytes (LB) and NK cells from human fresh heparinized peripheral blood. B cells and NK cells were gated from the lymphocytes gate. NK cells were defined as CD56^+^ CD3^-^ cells. NKT cells were defined as CD56^+^ CD3^+^ cells. T lymphocytes (LT) were defined as CD56^-^ CD19^-^ CD3^+^ cells. Naïve B cells were defined as CD19^+^ IgD^+^ CD27^-^ cells, memory B cells as CD19^+^ IgD^-^ CD27^+^ cells, and marginal zone (MZ) B cells as CD19^+^ IgD^+^ CD27^+^ cells. SS: side scatter, FS: Forward scatter.
(PDF)

S3 Fig Correlation between CD4^+^ CD25^+^ FoxP3^+^ Treg and CD4^+^ CD25^+^ CD127^-^ Treg. A. Representative flow cytometry of Tregs (red) using CD4^+^ CD25^+^ FoxP3^+^ gating, and the same Tregs (CD4^+^ CD25^+^ FoxP3^+^) (red) retro gated using CD25 and CD127 markers.
B. Correlation between CD4^+^ CD25^+^ FoxP3^+^ Treg and CD4^+^ CD25^+^ CD127^-^ Treg.
C. Concordance between CD4^+^ CD25^+^ FoxP3^+^ Treg and CD4^+^ CD25^+^ CD127^-^ Treg.
(PDF)

S4 Fig **B cells, NK cells and T cells gamma delta phenotyping** according to NT1 status (respectively A, B and C), and to H1N1 status in NT1 patients (respectively D, E and F).
(PDF)

S5 Fig Influence of NT1 disease duration and age on Tregs frequency and Tregs memory. Correlation between Tregs frequency and NT1 disease duration (A), between effector memory Tregs frequency and disease duration (B), and between effector memory Tregs frequency and age (C).
(PDF)
