# Supplementary material for: Narcolepsy Type 1 Is Associated with a Systemic Increase and Activation of Regulatory T Cells and with a Systemic Activation of Global T Cells
Source: PLoS One. 2017 Jan 20;12(1):e0169836. doi: 10.1371/journal.pone.0169836 (PMC5249232; doi:10.1371/journal.pone.0169836)
Supplement: S1 Fig — CD4+ and CD8+ T cells were gated from the lymphocytes gate. Naïve T cells were defined as CD45RA+ CD62L+ cells, central memory (CM) T cells as CD45RA- CD62L+, effector memory (EM) T cells as CD45RA- CD62L-, and terminally differentiated (TEMRA) T cells as CD45RA+ CD62L-. Activated memory Tregs (AM) were defined as CD45RA- HLA-DR+. (PDF) [file pone.0169836.s001.pdf]

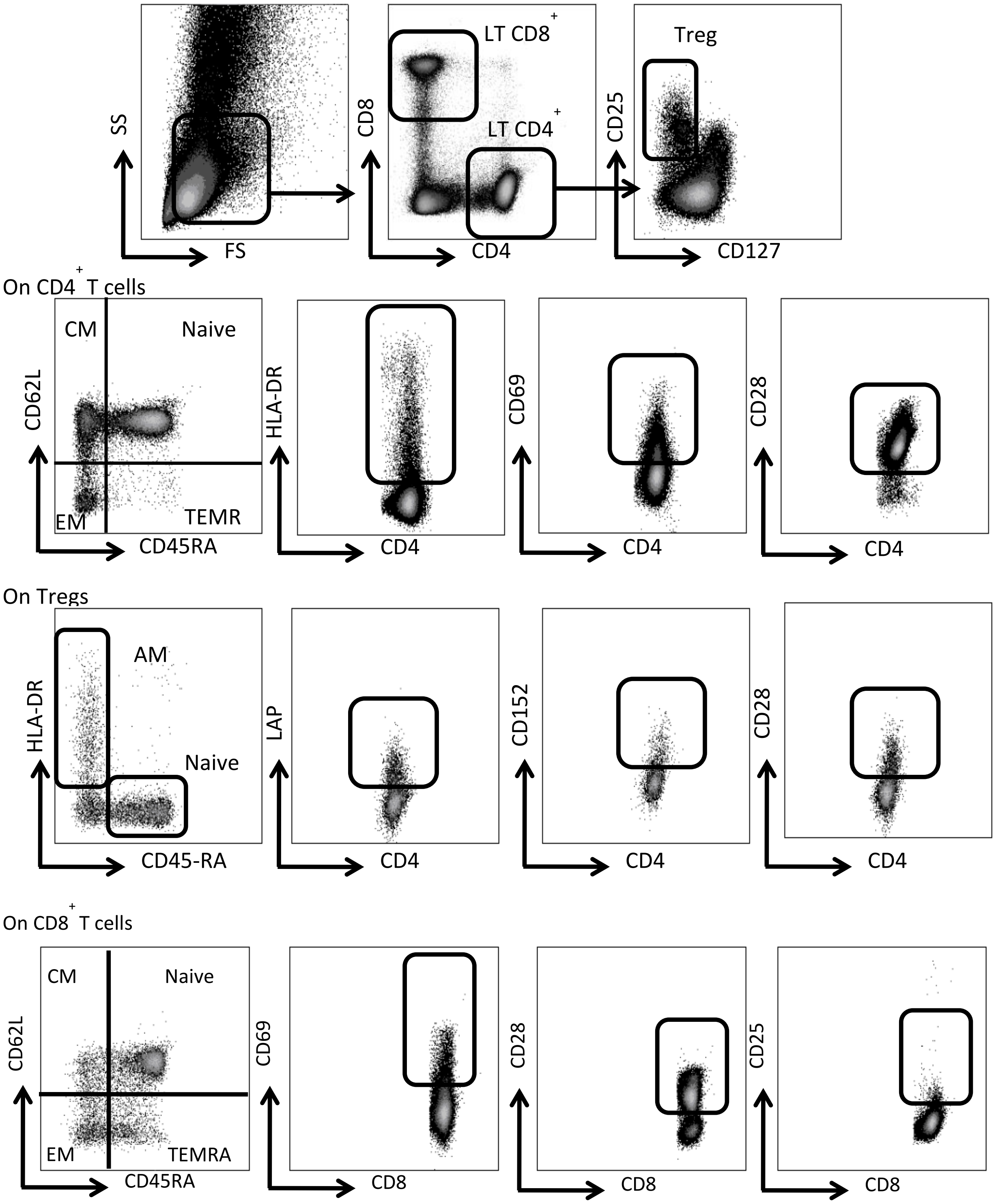

S1 Fig. Representative flow cytometry analysis of $CD4^+$ and $CD8^+$ T cells and of $CD4^+$ Tregs from human fresh heparinized peripheral blood. $CD4^+$ and $CD8^+$ T cells were gated from the lymphocytes gate. Naïve T cells were defined as $CD45RA^+$ $CD62L^+$ cells, central memory (CM) T cells as $CD45RA^-$ $CD62L^+$, effector memory (EM) T cells as $CD45RA^-$ $CD62L^-$, and terminally differentiated (TEMRA) T cells as $CD45RA^+$ $CD62L^-$. Activated memory Tregs (AM) were defined as $CD45RA^-$ HLA-DR^+.
