# Supplementary material for: Narcolepsy Type 1 Is Associated with a Systemic Increase and Activation of Regulatory T Cells and with a Systemic Activation of Global T Cells
Source: PLoS One. 2017 Jan 20;12(1):e0169836. doi: 10.1371/journal.pone.0169836 (PMC5249232; doi:10.1371/journal.pone.0169836)
Supplement: S2 Fig — B cells and NK cells were gated from the lymphocytes gate. NK cells were defined as CD56+ CD3- cells. NKT cells were defined as CD56+ CD3+ cells. T lymphocytes (LT) were defined as CD56- CD19- CD3+ cells. Naïve B cells were defined as CD19+ IgD+ CD27- cells, memory B cells as CD19+ IgD- CD27+ cells, and marginal zone (MZ) B cells as CD19+ IgD+ CD27+ cells. SS: side scatter, FS: Forward scatter. (PDF) [file pone.0169836.s002.pdf]

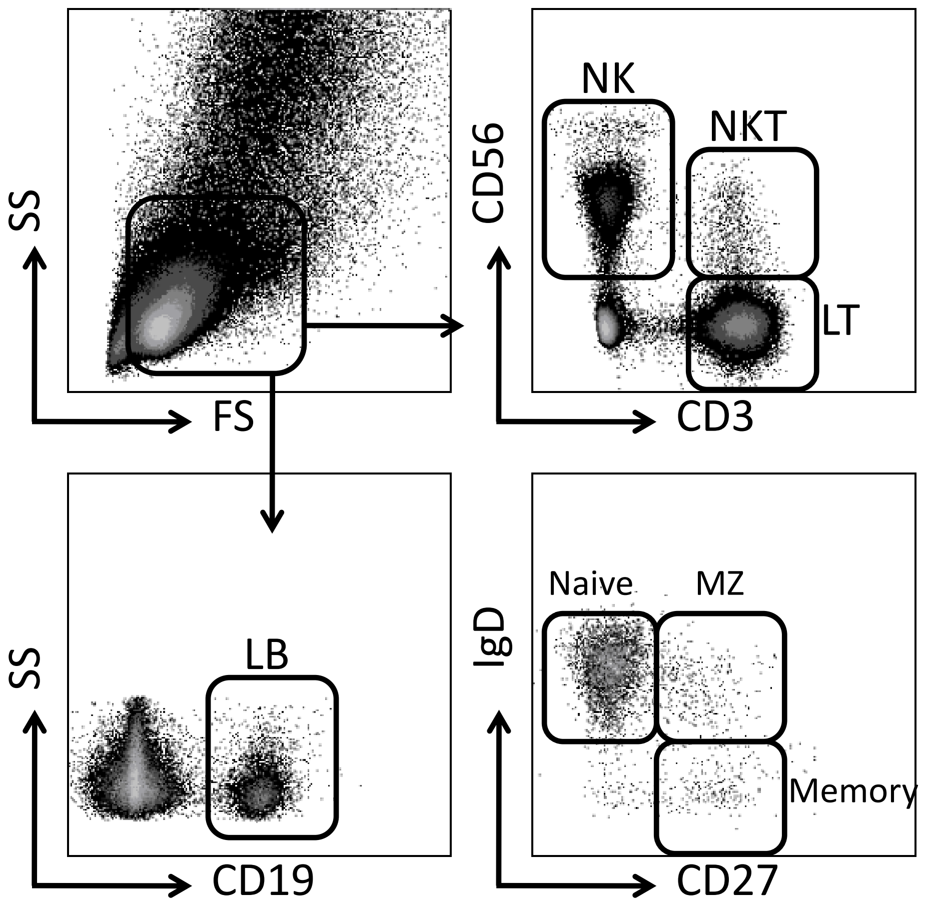

S2 Fig. Representative flow cytometry analysis of B lymphocytes (LB) and NK cells from human fresh heparinized peripheral blood. B cells and NK cells were gated from the lymphocytes gate. NK cells were defined as $CD56^+$ $CD3^-$ cells. NKT cells were defined as $CD56^+$ $CD3^+$ cells. T lymphocytes (LT) were defined as $CD56^-$ $CD19^-$ $CD3^+$ cells. Naïve B cells were defined as $CD19^+$ IgD^+ $CD27^-$ cells, memory B cells as $CD19^+$ IgD^- $CD27^+$ cells, and marginal zone (MZ) B cells as $CD19^+$ IgD^+ $CD27^+$ cells. SS: side scatter, FS: Forward scatter.
