# Supplementary material for: Narcolepsy Type 1 Is Associated with a Systemic Increase and Activation of Regulatory T Cells and with a Systemic Activation of Global T Cells
Source: PLoS One. 2017 Jan 20;12(1):e0169836. doi: 10.1371/journal.pone.0169836 (PMC5249232; doi:10.1371/journal.pone.0169836)
Supplement: S3 Fig — A. Representative flow cytometry of Tregs (red) using CD4+ CD25+ FoxP3+ gating, and the same Tregs (CD4+ CD25+ FoxP3+) (red) retro gated using CD25 and CD127 markers. B. Correlation between CD4+ CD25+ FoxP3+ Treg and CD4+ CD25+ CD127- Treg. C. Concordance between CD4+ CD25+ FoxP3+ Treg and CD4+ CD25+ CD127- Treg. (PDF) [file pone.0169836.s003.pdf]

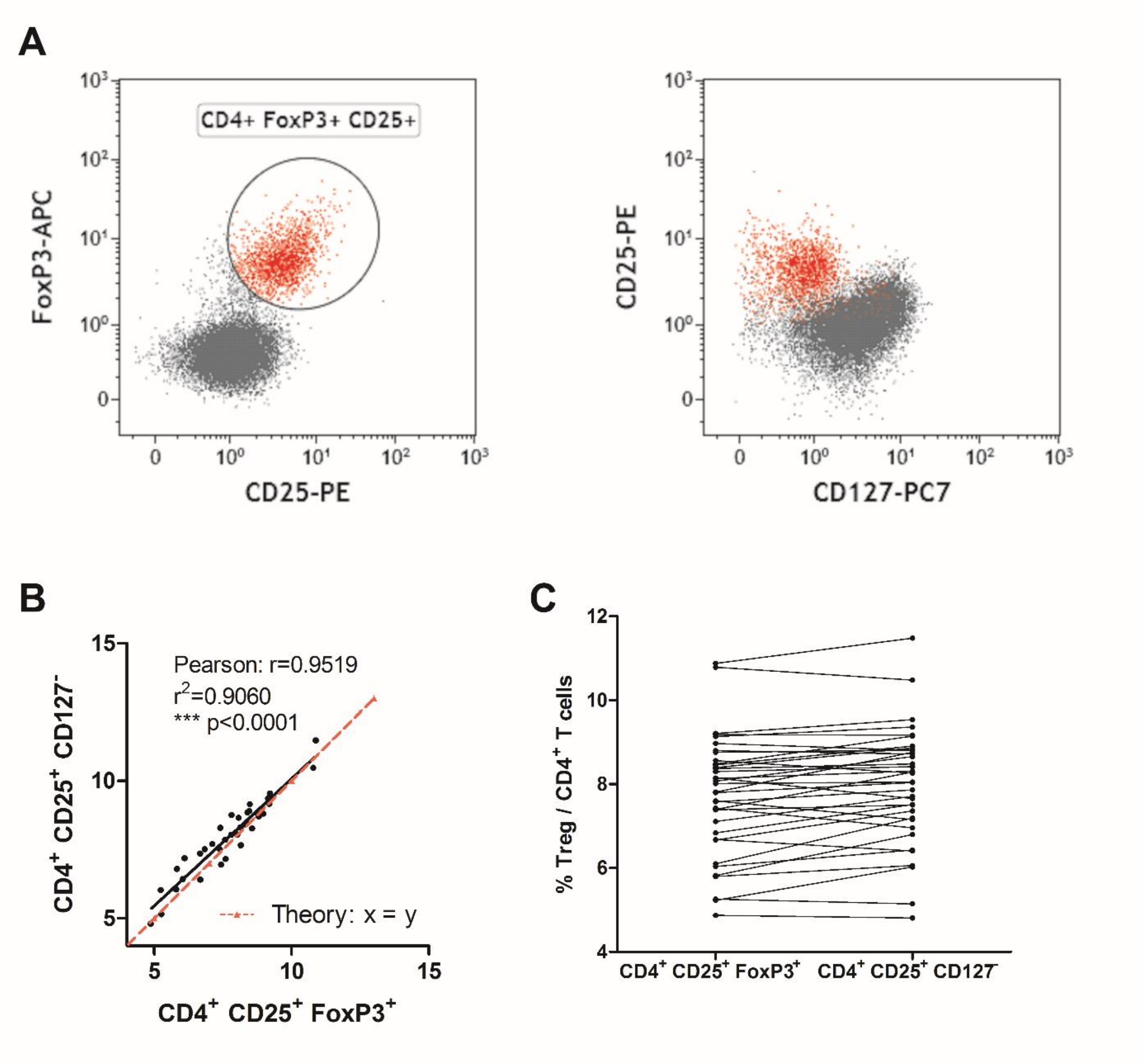

S3 Fig. correlation between CD4$^+$ CD25$^+$ FoxP3$^+$ Treg and CD4$^+$ CD25$^+$ CD127$^-$ Treg

A. Representative flow cytometry of Tregs (red) using CD4$^+$ CD25$^+$ FoxP3$^+$ gating, and the same Tregs (CD4$^+$ CD25$^+$ FoxP3$^+$) (red) retro gated using CD25 and CD127 markers.

B. Correlation between CD4$^+$ CD25$^+$ FoxP3$^+$ Treg and CD4$^+$ CD25$^+$ CD127$^-$ Treg

C. Concordance between CD4$^+$ CD25$^+$ FoxP3$^+$ Treg and CD4$^+$ CD25$^+$ CD127$^-$ Treg
