# Supplementary material for: Narcolepsy Type 1 Is Associated with a Systemic Increase and Activation of Regulatory T Cells and with a Systemic Activation of Global T Cells
Source: PLoS One. 2017 Jan 20;12(1):e0169836. doi: 10.1371/journal.pone.0169836 (PMC5249232; doi:10.1371/journal.pone.0169836)
Supplement: S4 Fig — B cells, NK cells and T cells gamma delta phenotyping according to NT1 status (respectively A, B and C), and to H1N1 status in NT1 patients (respectively D, E and F). (PDF) [file pone.0169836.s004.pdf]

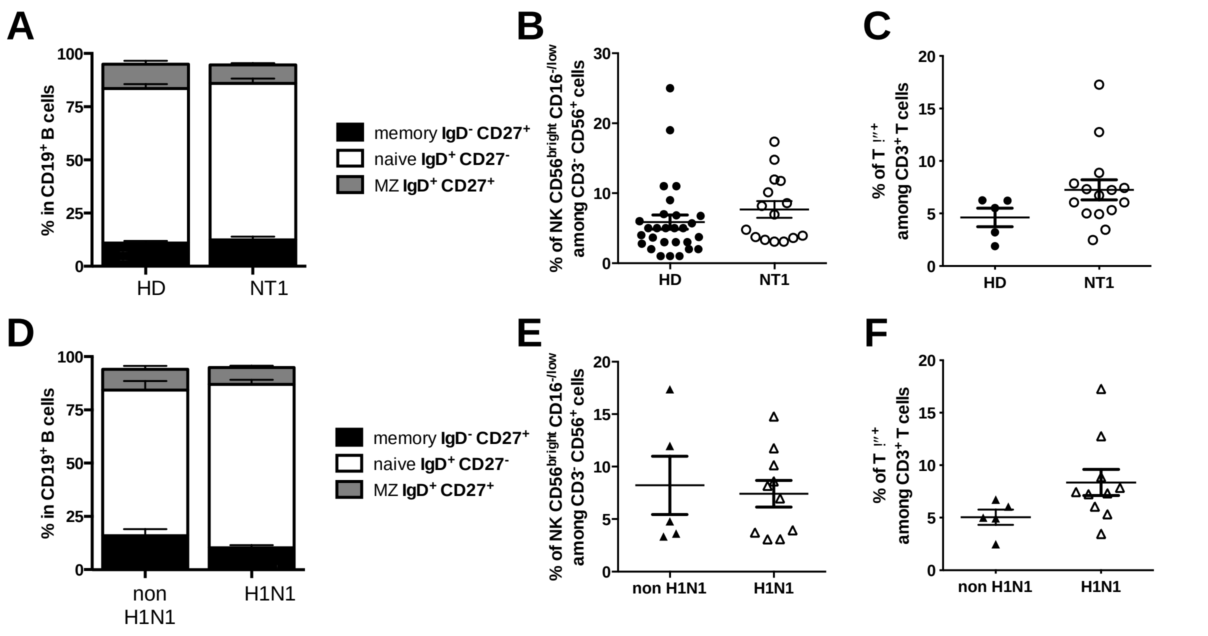

S4 Fig. B cells, NK cells and T cells gamma delta phenotyping according to NT1 status (respectively A, B and C), and to H1N1 status in NT1 patients (respectively D, E and F).
