# Supplementary material for: Narcolepsy Type 1 Is Associated with a Systemic Increase and Activation of Regulatory T Cells and with a Systemic Activation of Global T Cells
Source: PLoS One. 2017 Jan 20;12(1):e0169836. doi: 10.1371/journal.pone.0169836 (PMC5249232; doi:10.1371/journal.pone.0169836)
Supplement: S5 Fig — Correlation between Tregs frequency and NT1 disease duration (A), between effector memory Tregs frequency and disease duration (B), and between effector memory Tregs frequency and age (C). (PDF) [file pone.0169836.s005.pdf]

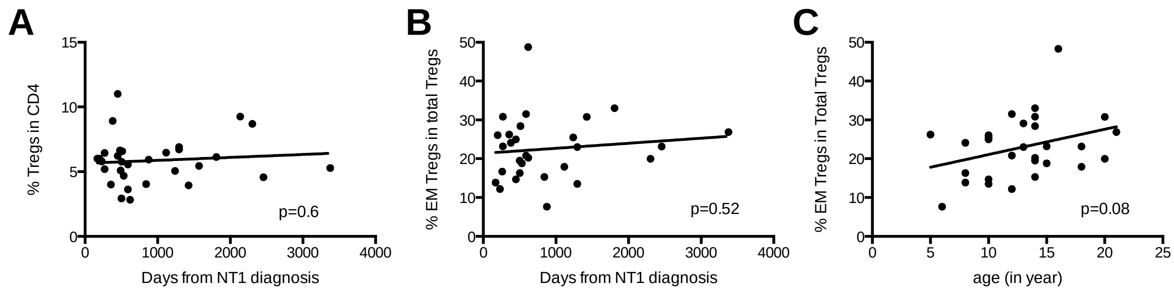

S5 Fig. Influence of NT1 disease duration and age on Tregs frequency and Tregs memory. Correlation between Tregs frequency and NT1 disease duration (A), between effector memory Tregs frequency and disease duration (B), and between effector memory Tregs frequency and age (C).
